# Supplementary material for: A yeast phenomic model for the influence of Warburg metabolism on genetic buffering of doxorubicin
Source: Cancer Metab. 2019 Oct 23;7:9. doi: 10.1186/s40170-019-0201-3 (PMC6806529; doi:10.1186/s40170-019-0201-3)
Supplement: Supplementary file 9 — Additional file 9. GO term-specific heatmaps for REMc/GTF-enriched clusters. GO term-specific heatmaps for significant GO process terms were generated as described in methods and Figs. 3 and 4. Any related child terms are presented in subsequent pages of the parent file name. GO terms with more than 100 children, with 2 or fewer genes annotated to the term, or a file size over 300KB are not shown. All heatmaps are generated with the same layout (see Figs. 3 and 4). [file 40170_2019_201_MOESM9_ESM.bz2 › Additional_File9_GOTermHeatmaps/Additional_File9_GOTermHeatmap/protein_targeting.pdf]

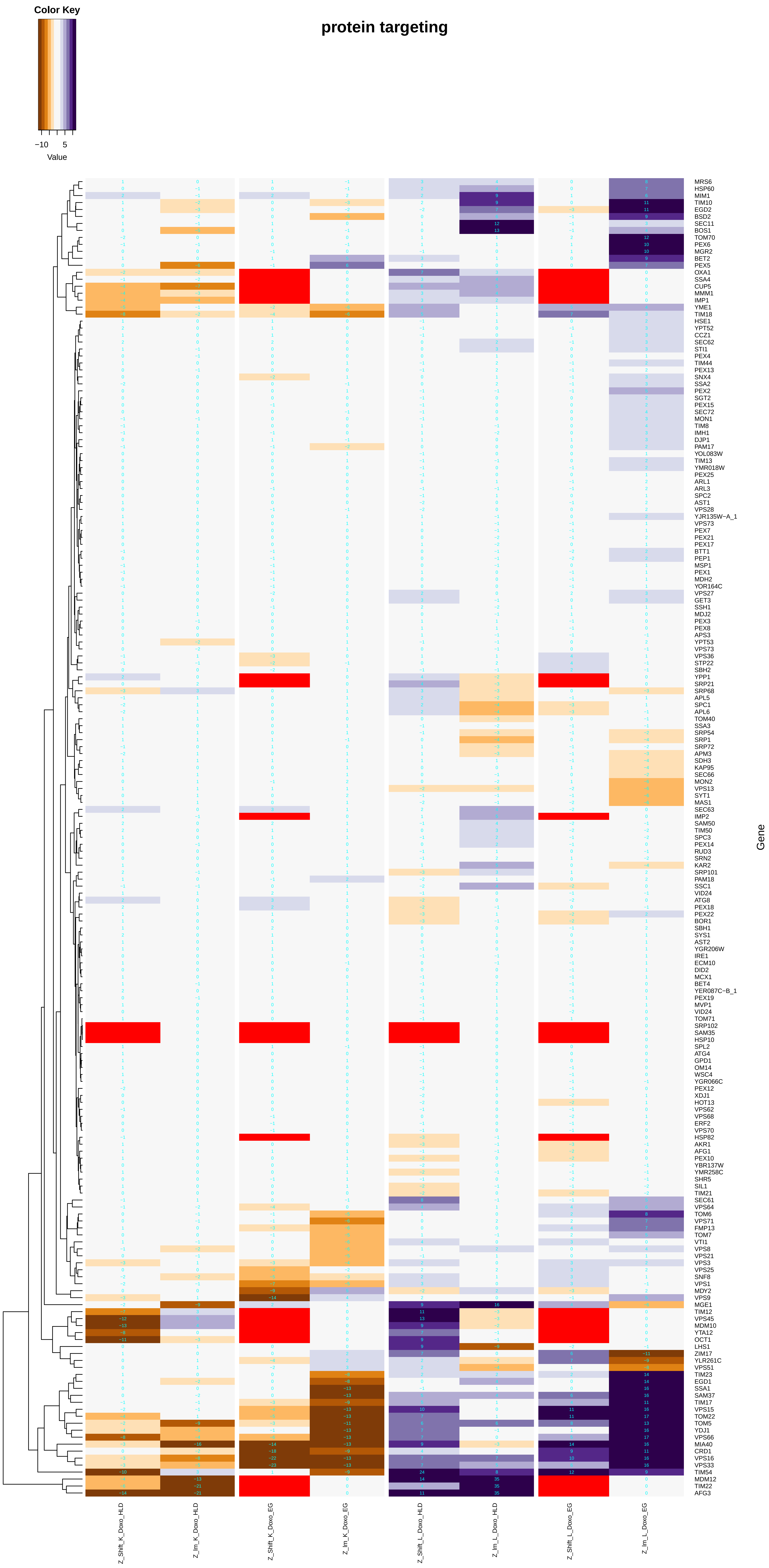

protein targeting
Color Key
−10
5
Value
MRS6
HSP60
MIM1
TIM10
EGD2
BSD2
SEC11
BOS1
TOM70
PEX6
MGR2
BET2
PEX5
OXA1
SSA4
CUP5
MMM1
IMP1
YME1
TIM18
HSE1
YPT52
CCZ1
SEC62
STI1
PEX4
TIM44
PEX13
SNX4
SSA2
PEX2
SGT2
PEX15
SEC72
MON1
TIM8
IMH1
DJP1
PAM17
YOL083W
TIM13
YMR018W
PEX25
ARL1
ARL3
SPC2
AST1
VPS28
YJR135W−A_1
VPS73
PEX7
PEX21
PEX17
BTT1
PEP1
MSP1
PEX1
MDH2
YOR164C
VPS27
GET3
SSH1
MDJ2
PEX3
PEX8
APS3
YPT53
VPS73
VPS36
STP22
SBH2
YPP1
SRP21
SRP68
APL5
SPC1
APL6
TOM40
SSA3
SRP54
SRP1
SRP72
APM3
SDH3
KAP95
SEC66
MON2
VPS13
SYT1
MAS1
SEC63
IMP2
SAM50
TIM50
SPC3
PEX14
RUD3
SRN2
KAR2
SRP101
PAM18
SSC1
VID24
ATG8
PEX18
PEX22
BOR1
SBH1
SYS1
AST2
YGR206W
IRE1
ECM10
DID2
MCX1
BET4
YER087C−B_1
PEX19
MVP1
VID24
TOM71
SRP102
SAM35
HSP10
SPL2
ATG4
GPD1
OM14
WSC4
YGR066C
PEX12
XDJ1
HOT13
VPS62
VPS68
ERF2
VPS70
HSP82
AKR1
AFG1
PEX10
YBR137W
YMR258C
SHR5
SIL1
TIM21
SEC61
VPS64
TOM6
VPS71
FMP13
TOM7
VTI1
VPS8
VPS21
VPS3
VPS25
SNF8
VPS1
MDY2
VPS9
MGE1
TIM12
VPS45
MDM10
YTA12
OCT1
LHS1
ZIM17
YLR261C
VPS51
TIM23
EGD1
SSA1
SAM37
TIM17
VPS15
TOM22
TOM5
YDJ1
VPS66
MIA40
CRD1
VPS16
VPS33
TIM54
MDM12
TIM22
AFG3
Gene
Z_Shift_K_Doxo_HLD
Z_Im_K_Doxo_HLD
Z_Shift_K_Doxo_EG
Z_Im_K_Doxo_EG
Z_Shift_L_Doxo_HLD
Z_Im_L_Doxo_HLD
Z_Shift_L_Doxo_EG
Z_Im_L_Doxo_EG

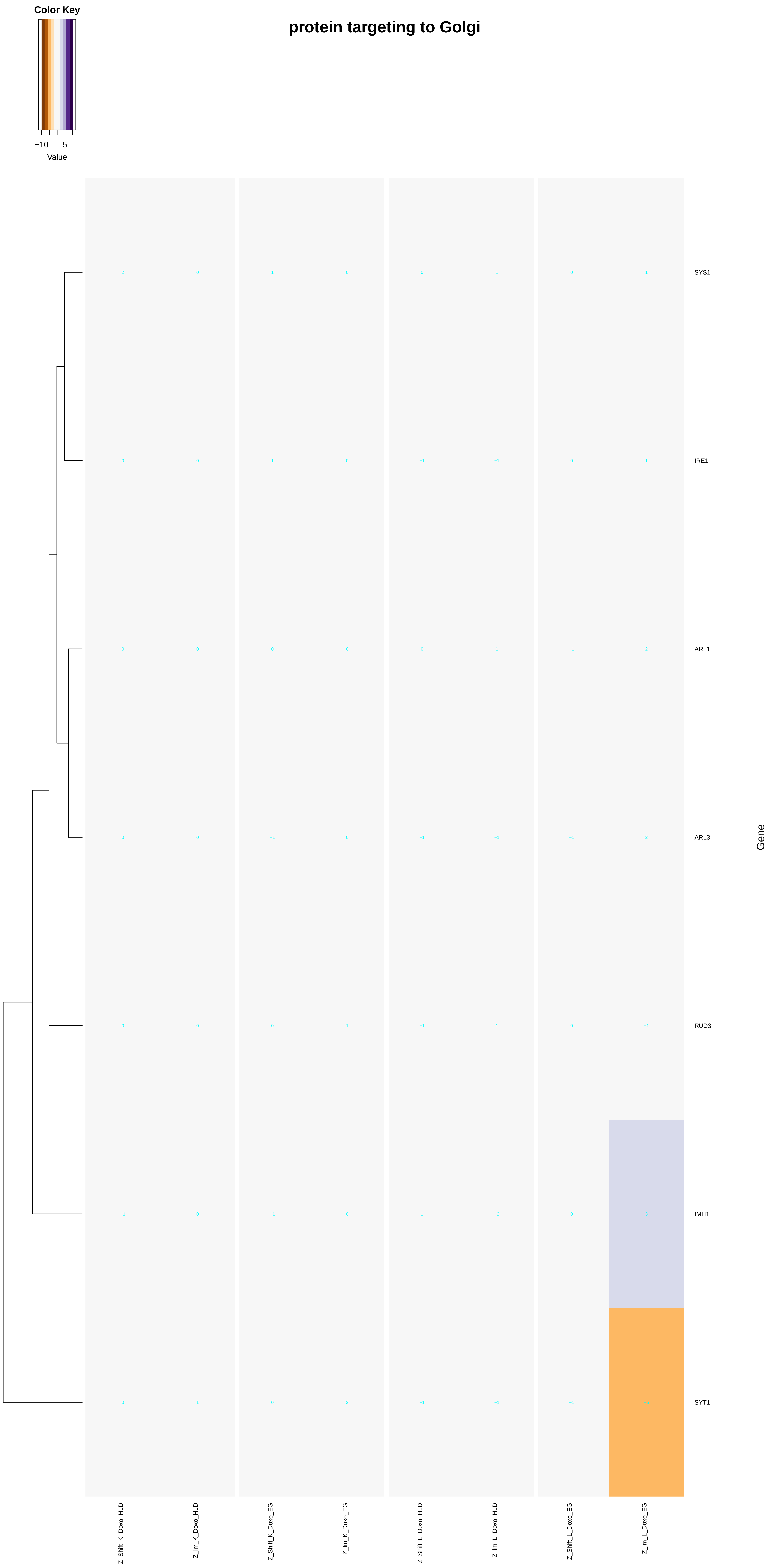

Color Key
−10
5
Value
protein targeting to Golgi
SYS1
IRE1
ARL1
ARL3
RUD3
IMH1
SYT1
Gene
Z_Shift_K_Doxo_HLD
Z_lm_K_Doxo_HLD
Z_Shift_K_Doxo_EG
Z_lm_K_Doxo_EG
Z_Shift_L_Doxo_HLD
Z_lm_L_Doxo_HLD
Z_Shift_L_Doxo_EG
Z_lm_L_Doxo_EG

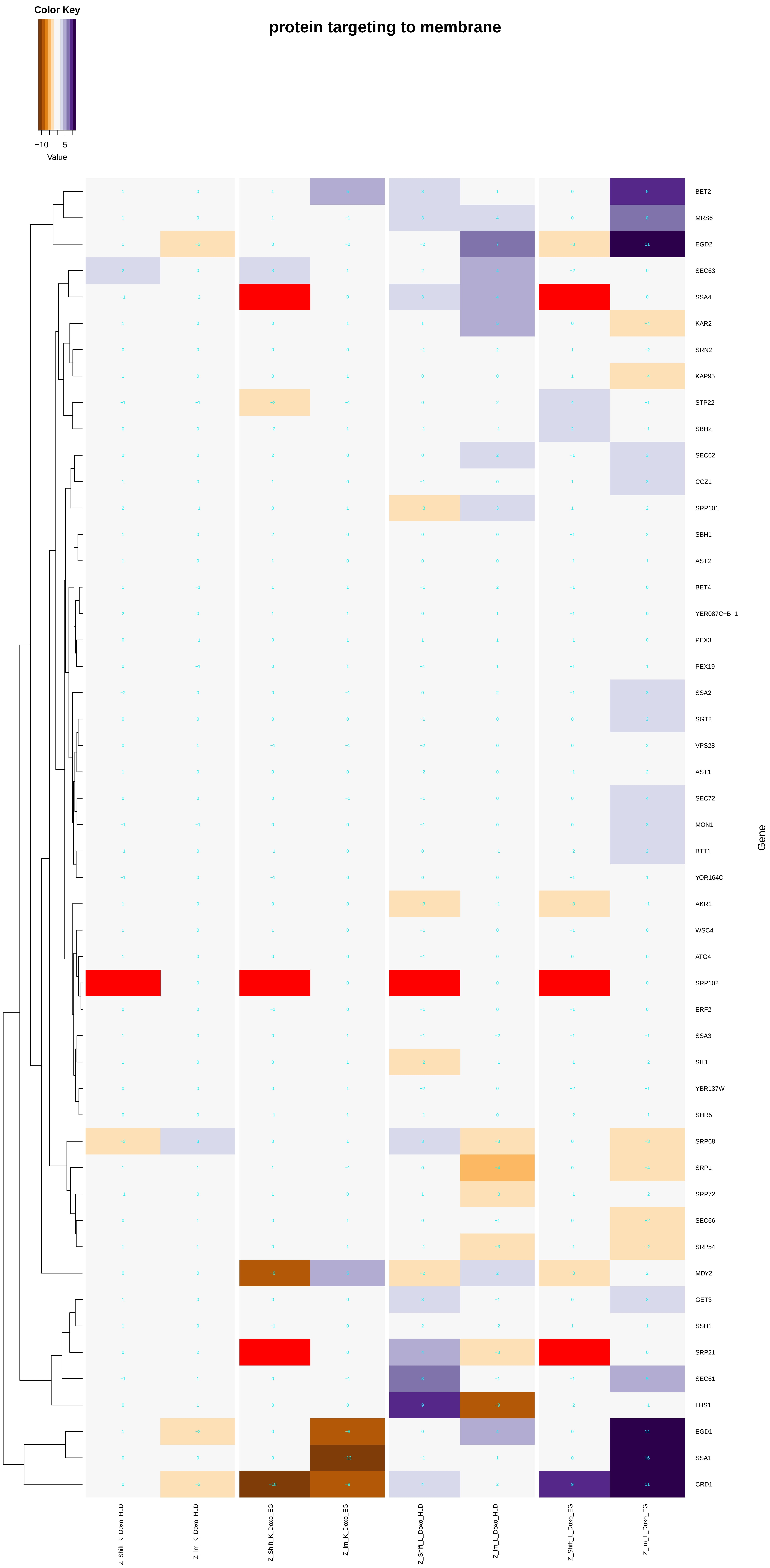
protein targeting to membrane
Color Key
−10
5
Value
Gene
BET2
MRS6
EGD2
SEC63
SSA4
KAR2
SRN2
KAP95
STP22
SBH2
SEC62
CCZ1
SRP101
SBH1
AST2
BET4
YER087C−B_1
PEX3
PEX19
SSA2
SGT2
VPS28
AST1
SEC72
MON1
BTT1
YOR164C
AKR1
WSC4
ATG4
SRP102
ERF2
SSA3
SIL1
YBR137W
SHR5
SRP68
SRP1
SRP72
SEC66
SRP54
MDY2
GET3
SSH1
SRP21
SEC61
LHS1
EGD1
SSA1
CRD1
Z_Shift_K_Doxo_HLD
Z_lm_K_Doxo_HLD
Z_Shift_K_Doxo_EG
Z_lm_K_Doxo_EG
Z_Shift_L_Doxo_HLD
Z_lm_L_Doxo_HLD
Z_Shift_L_Doxo_EG
Z_lm_L_Doxo_EG

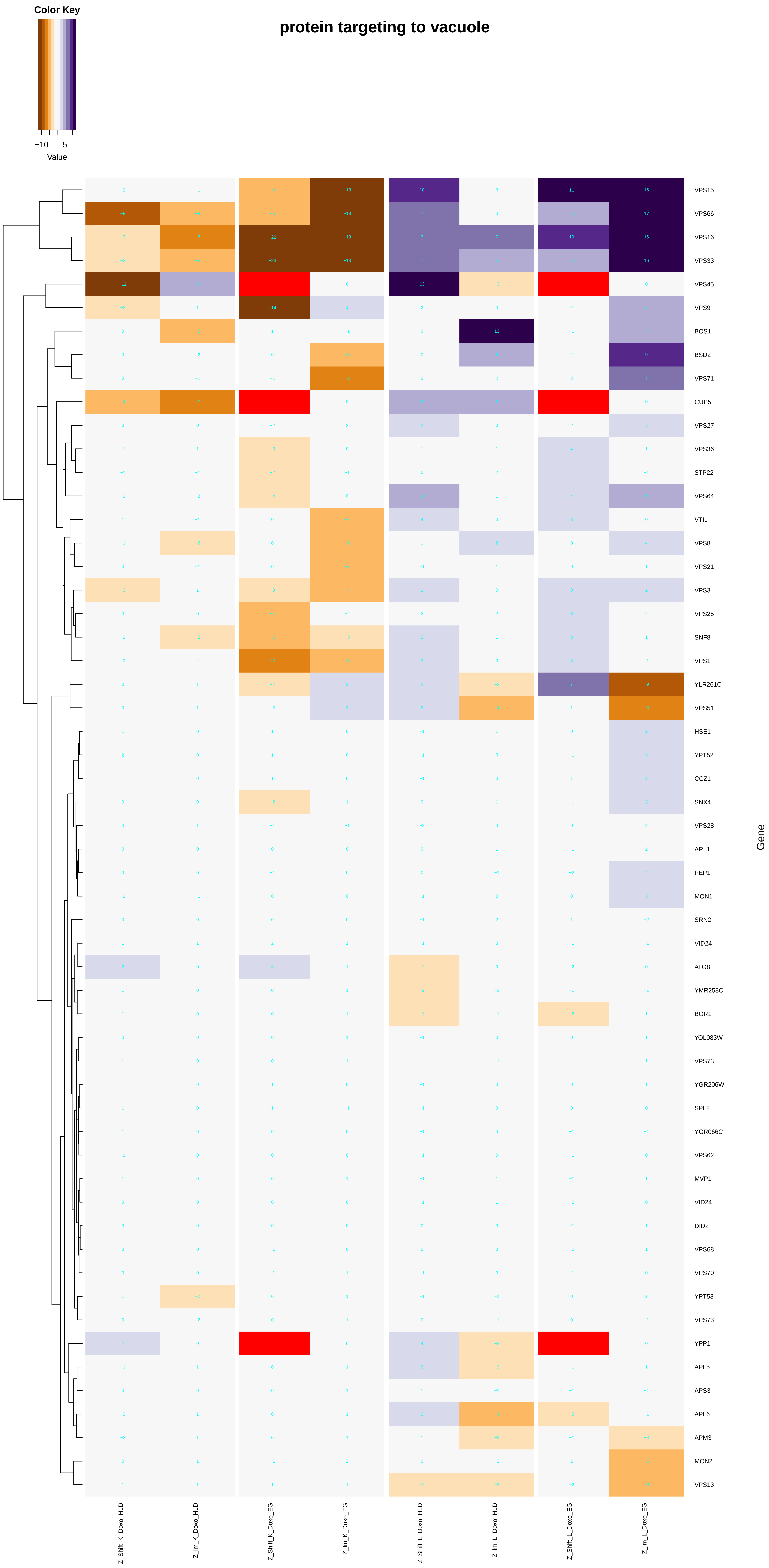
protein targeting to vacuole
Color Key
−10
5
Value
VPS15
VPS66
VPS16
VPS33
VPS45
VPS9
BOS1
BSD2
VPS71
CUP5
VPS27
VPS36
STP22
VPS64
VTI1
VPS8
VPS21
VPS3
VPS25
SNF8
VPS1
YLR261C
VPS51
HSE1
YPT52
CCZ1
SNX4
VPS28
ARL1
PEP1
MON1
SRN2
VID24
ATG8
YMR258C
BOR1
YOL083W
VPS73
YGR206W
SPL2
YGR066C
VPS62
MVP1
VID24
DID2
VPS68
VPS70
YPT53
VPS73
YPP1
APL5
APS3
APL6
APM3
MON2
VPS13
Gene
Z_Shift_K_Doxo_HLD
Z_lm_K_Doxo_HLD
Z_Shift_K_Doxo_EG
Z_lm_K_Doxo_EG
Z_Shift_L_Doxo_HLD
Z_lm_L_Doxo_HLD
Z_Shift_L_Doxo_EG
Z_lm_L_Doxo_EG

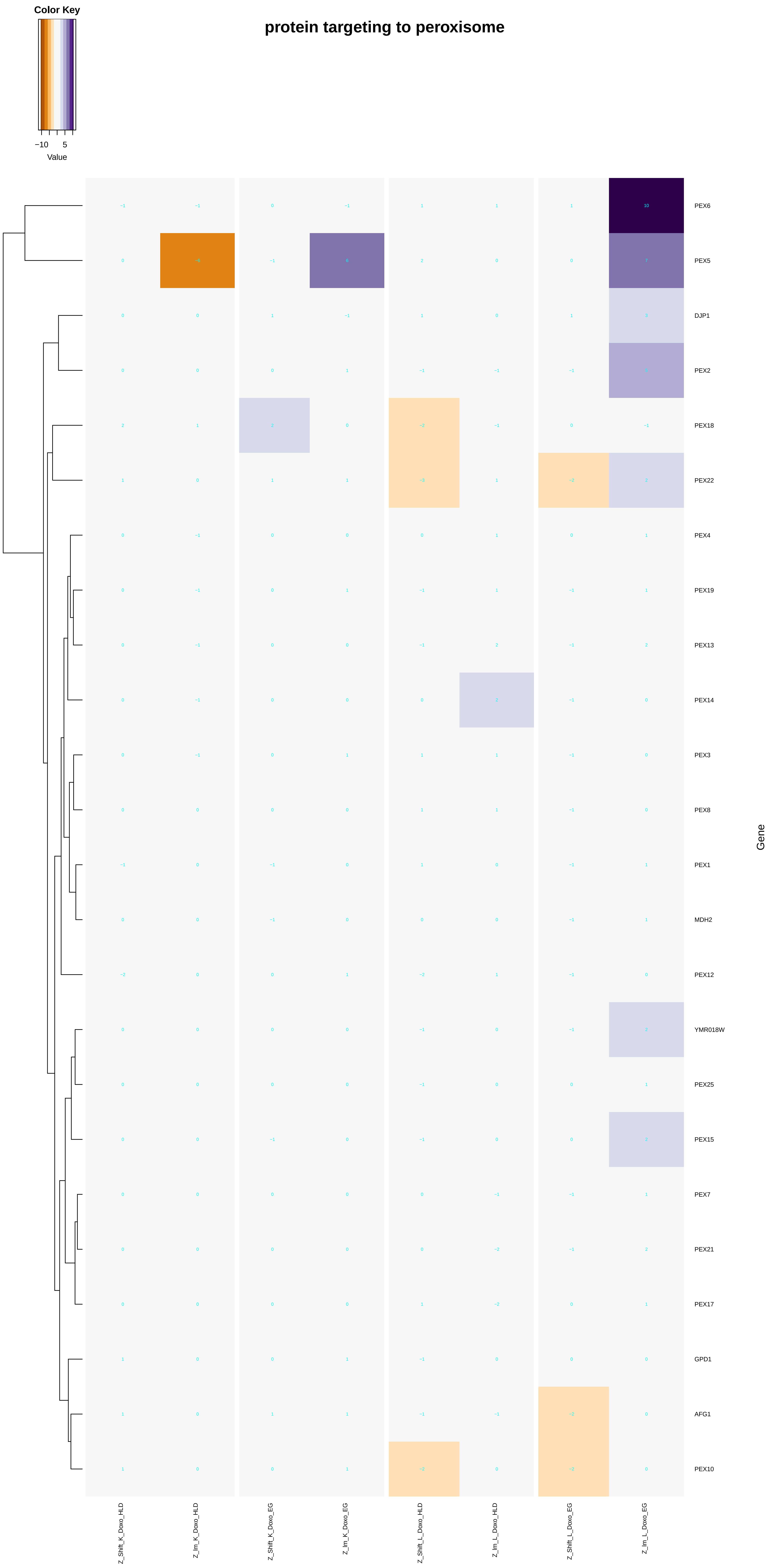
Color Key
−10
5
Value
protein targeting to peroxisome
PEX6
PEX5
DJP1
PEX2
PEX18
PEX22
PEX4
PEX19
PEX13
PEX14
PEX3
PEX8
PEX1
MDH2
PEX12
YMR018W
PEX25
PEX15
PEX7
PEX21
PEX17
GPD1
AFG1
PEX10
Gene
Z_Shift_K_Doxo_HLD
Z_lm_K_Doxo_HLD
Z_Shift_K_Doxo_EG
Z_lm_K_Doxo_EG
Z_Shift_L_Doxo_HLD
Z_lm_L_Doxo_HLD
Z_Shift_L_Doxo_EG
Z_lm_L_Doxo_EG

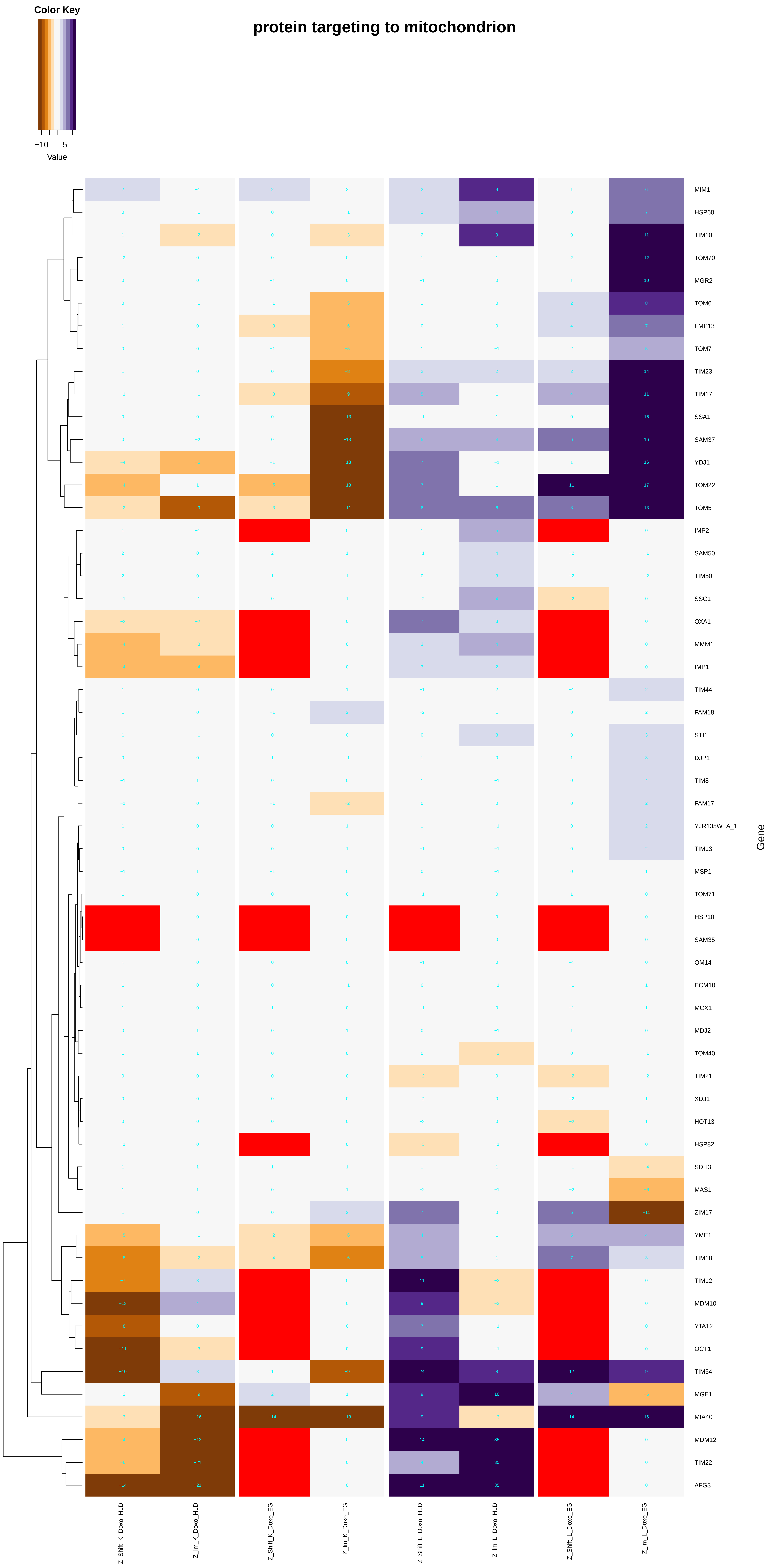

Color Key
−10
5
Value
protein targeting to mitochondrion
MIM1
HSP60
TIM10
TOM70
MGR2
TOM6
FMP13
TOM7
TIM23
TIM17
SSA1
SAM37
YDJ1
TOM22
TOM5
IMP2
SAM50
TIM50
SSC1
OXA1
MMM1
IMP1
TIM44
PAM18
STI1
DJP1
TIM8
PAM17
YJR135W−A_1
TIM13
MSP1
TOM71
HSP10
SAM35
OM14
ECM10
MCX1
MDJ2
TOM40
TIM21
XDJ1
HOT13
HSP82
SDH3
MAS1
ZIM17
YME1
TIM18
TIM12
MDM10
YTA12
OCT1
TIM54
MGE1
MIA40
MDM12
TIM22
AFG3
Gene
Z_Shift_K_Doxo_HLD
Z_Im_K_Doxo_HLD
Z_Shift_K_Doxo_EG
Z_Im_K_Doxo_EG
Z_Shift_L_Doxo_HLD
Z_Im_L_Doxo_HLD
Z_Shift_L_Doxo_EG
Z_Im_L_Doxo_EG

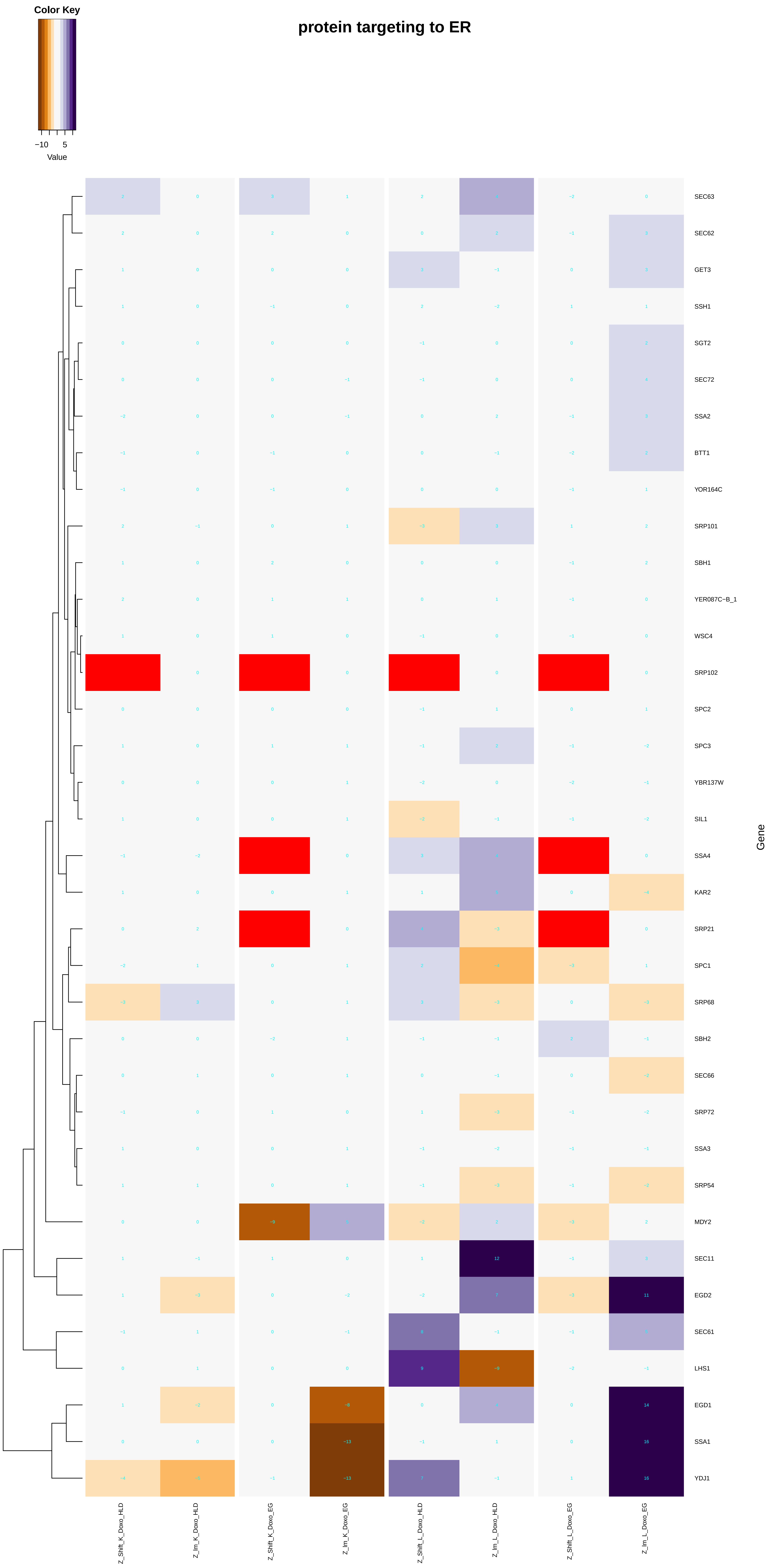
Color Key
−10
5
Value
protein targeting to ER
SEC63
SEC62
GET3
SSH1
SGT2
SEC72
SSA2
BTT1
YOR164C
SRP101
SBH1
YER087C−B_1
WSC4
SRP102
SPC2
SPC3
YBR137W
SIL1
SSA4
KAR2
SRP21
SPC1
SRP68
SBH2
SEC66
SRP72
SSA3
SRP54
MDY2
SEC11
EGD2
SEC61
LHS1
EGD1
SSA1
YDJ1
Gene
Z_Shift_K_Doxo_HLD
Z_Im_K_Doxo_HLD
Z_Shift_K_Doxo_EG
Z_Im_K_Doxo_EG
Z_Shift_L_Doxo_HLD
Z_Im_L_Doxo_HLD
Z_Shift_L_Doxo_EG
Z_Im_L_Doxo_EG

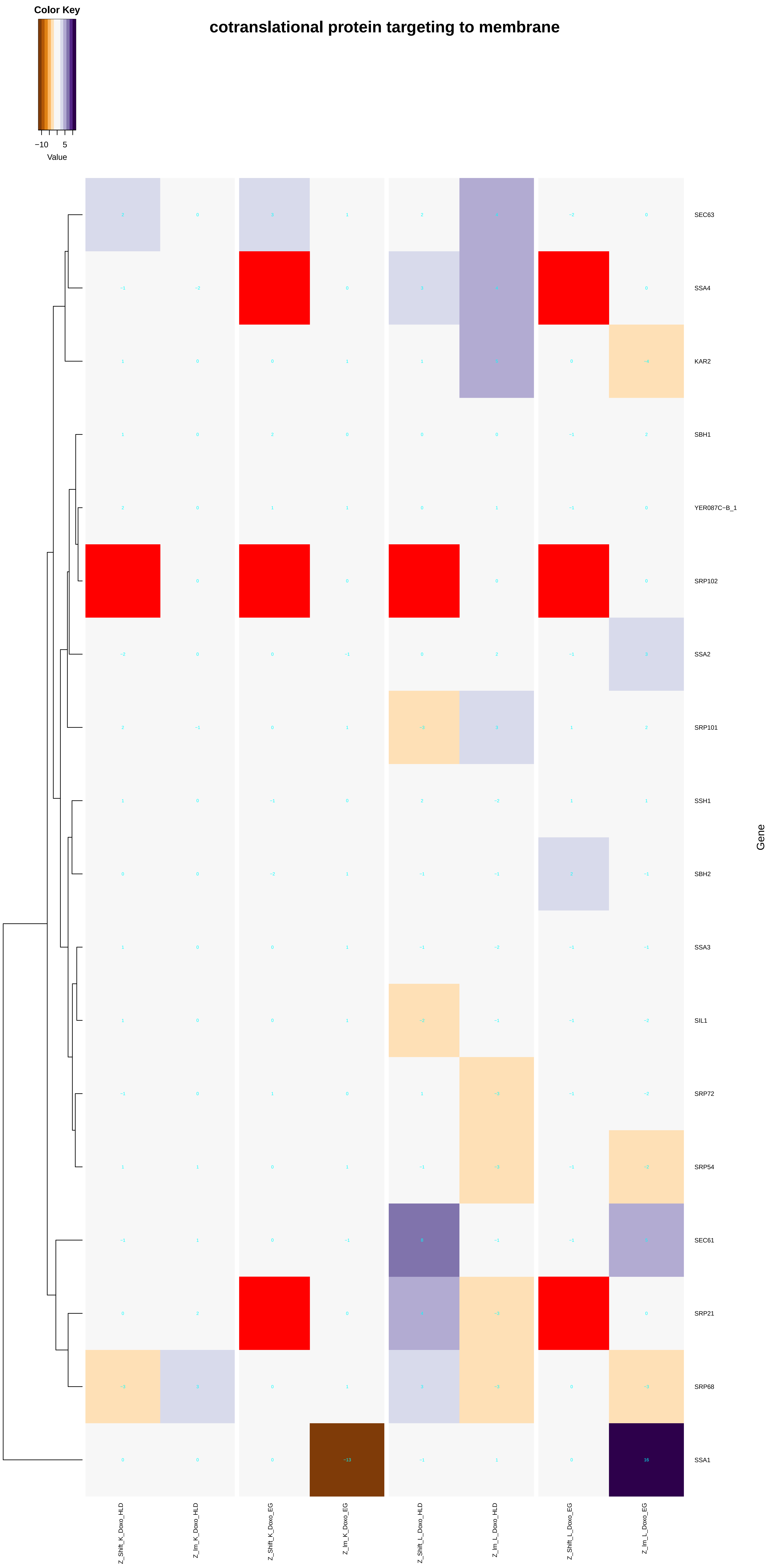
cotranslational protein targeting to membrane
Color Key
−10
5
Value
SEC63
SSA4
KAR2
SBH1
YER087C−B_1
SRP102
SSA2
SRP101
SSH1
SBH2
SSA3
SIL1
SRP72
SRP54
SEC61
SRP21
SRP68
SSA1
Gene
Z_Shift_K_Doxo_HLD
Z_Im_K_Doxo_HLD
Z_Shift_K_Doxo_EG
Z_Im_K_Doxo_EG
Z_Shift_L_Doxo_HLD
Z_Im_L_Doxo_HLD
Z_Shift_L_Doxo_EG
Z_Im_L_Doxo_EG

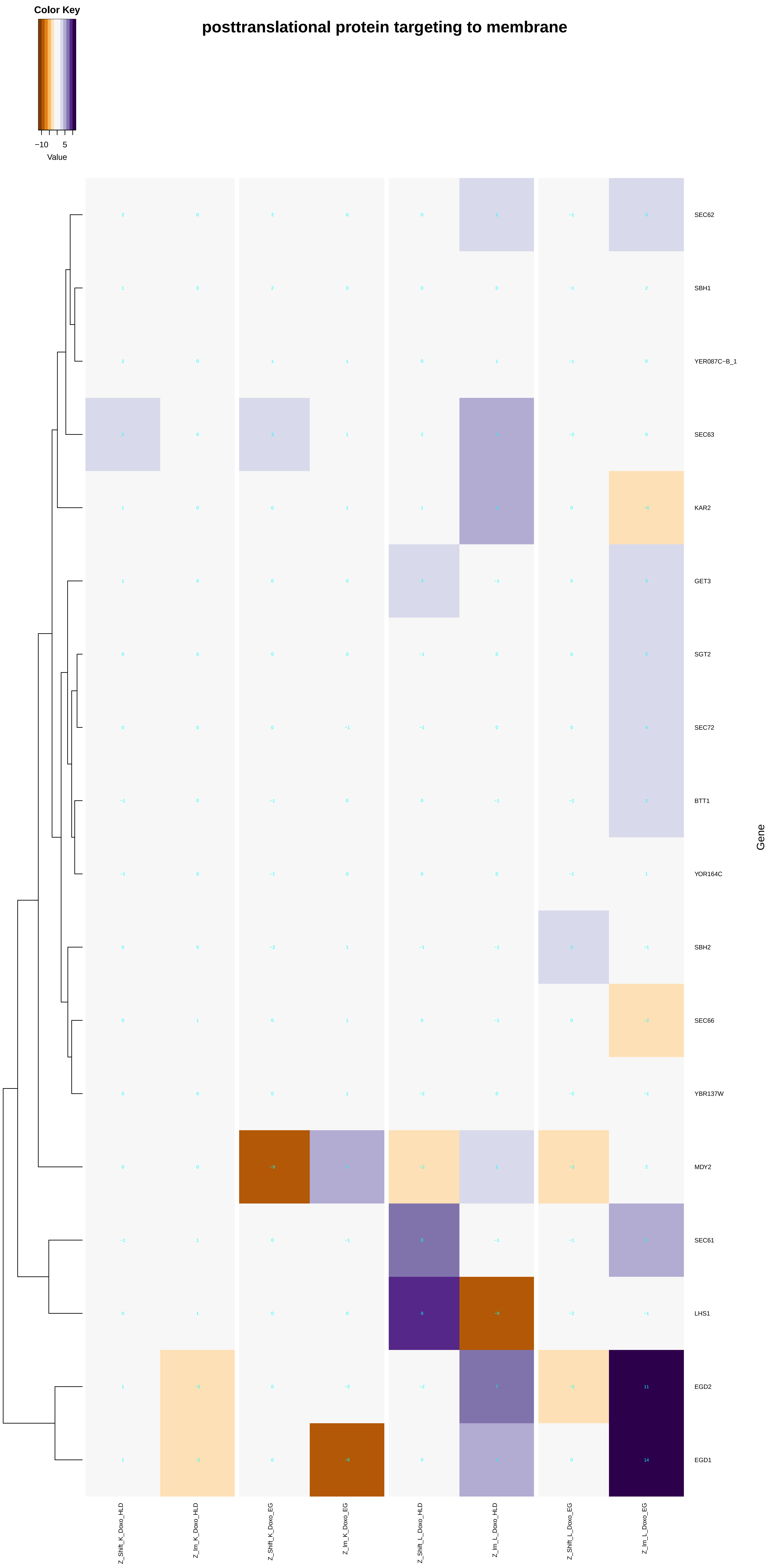

Color Key
−10
5
Value
posttranslational protein targeting to membrane
SEC62
SBH1
YER087C−B_1
SEC63
KAR2
GET3
SGT2
SEC72
BTT1
YOR164C
SBH2
SEC66
YBR137W
MDY2
SEC61
LHS1
EGD2
EGD1
Gene
Z_Shift_K_Doxo_HLD
Z_Im_K_Doxo_HLD
Z_Shift_K_Doxo_EG
Z_Im_K_Doxo_EG
Z_Shift_L_Doxo_HLD
Z_Im_L_Doxo_HLD
Z_Shift_L_Doxo_EG
Z_Im_L_Doxo_EG

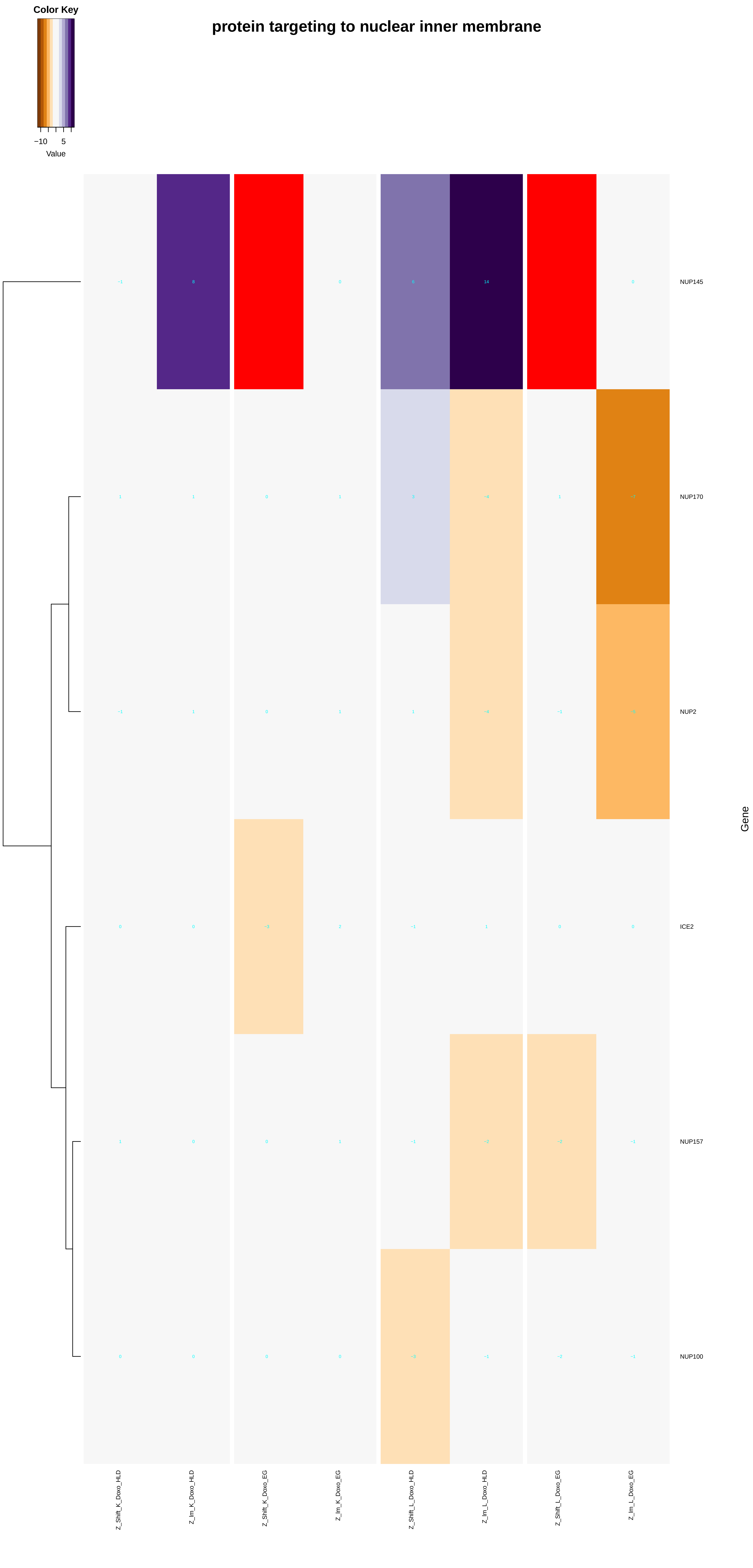

protein targeting to nuclear inner membrane
Color Key
−10
5
Value
NUP145
NUP170
NUP2
ICE2
NUP157
NUP100
Gene
Z_Shift_K_Doxo_HLD
Z_Im_K_Doxo_HLD
Z_Shift_K_Doxo_EG
Z_Im_K_Doxo_EG
Z_Shift_L_Doxo_HLD
Z_Im_L_Doxo_HLD
Z_Shift_L_Doxo_EG
Z_Im_L_Doxo_EG

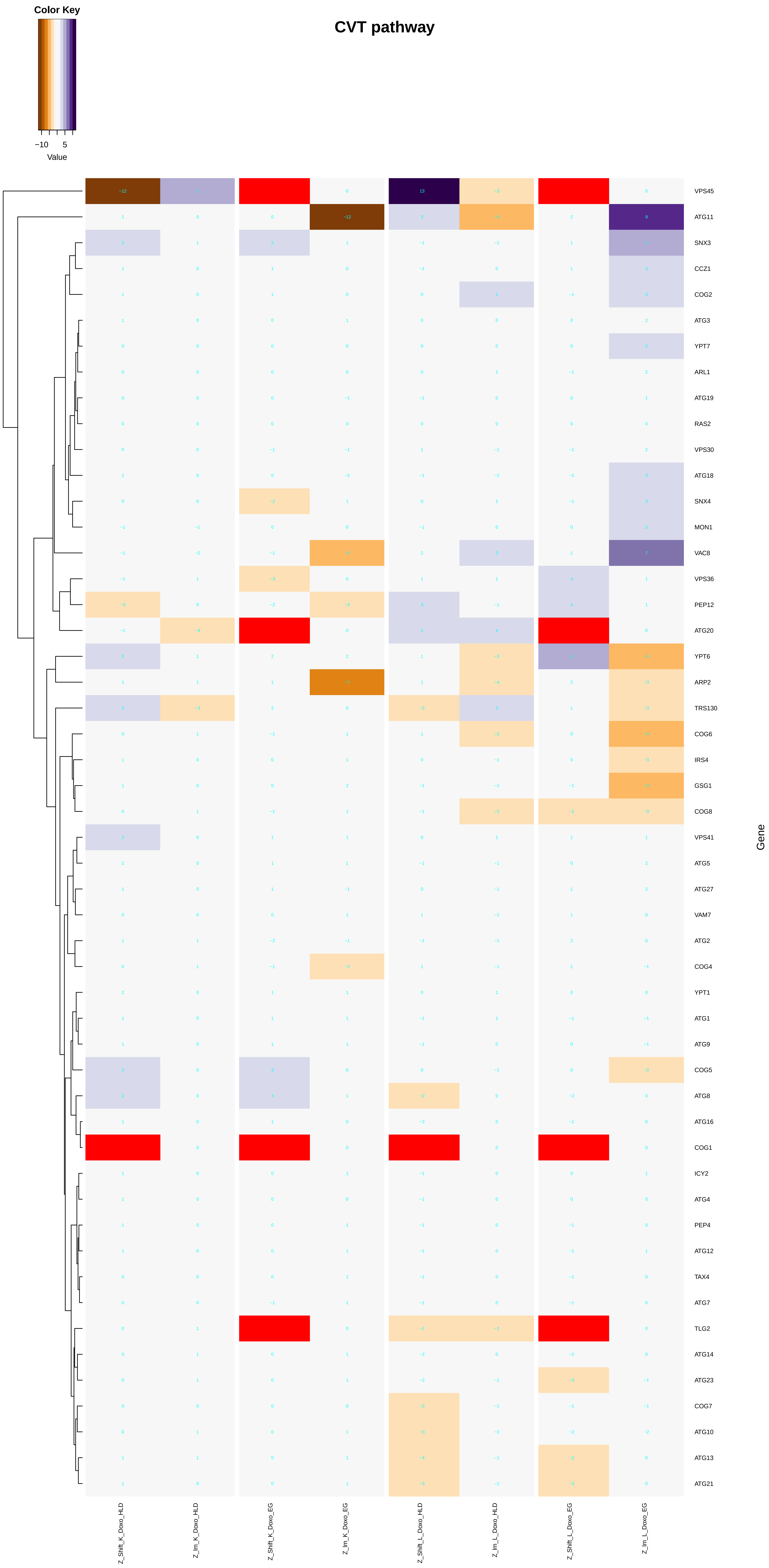

Color Key
−10
5
Value
CVT pathway
VPS45
ATG11
SNX3
CCZ1
COG2
ATG3
YPT7
ARL1
ATG19
RAS2
VPS30
ATG18
SNX4
MON1
VAC8
VPS36
PEP12
ATG20
YPT6
ARP2
TRS130
COG6
IRS4
GSG1
COG8
VPS41
ATG5
ATG27
VAM7
ATG2
COG4
YPT1
ATG1
ATG9
COG5
ATG8
ATG16
COG1
ICY2
ATG4
PEP4
ATG12
TAX4
ATG7
TLG2
ATG14
ATG23
COG7
ATG10
ATG13
ATG21
Gene
Z_Shift_K_Doxo_HLD
Z_Im_K_Doxo_HLD
Z_Shift_K_Doxo_EG
Z_Im_K_Doxo_EG
Z_Shift_L_Doxo_HLD
Z_Im_L_Doxo_HLD
Z_Shift_L_Doxo_EG
Z_Im_L_Doxo_EG

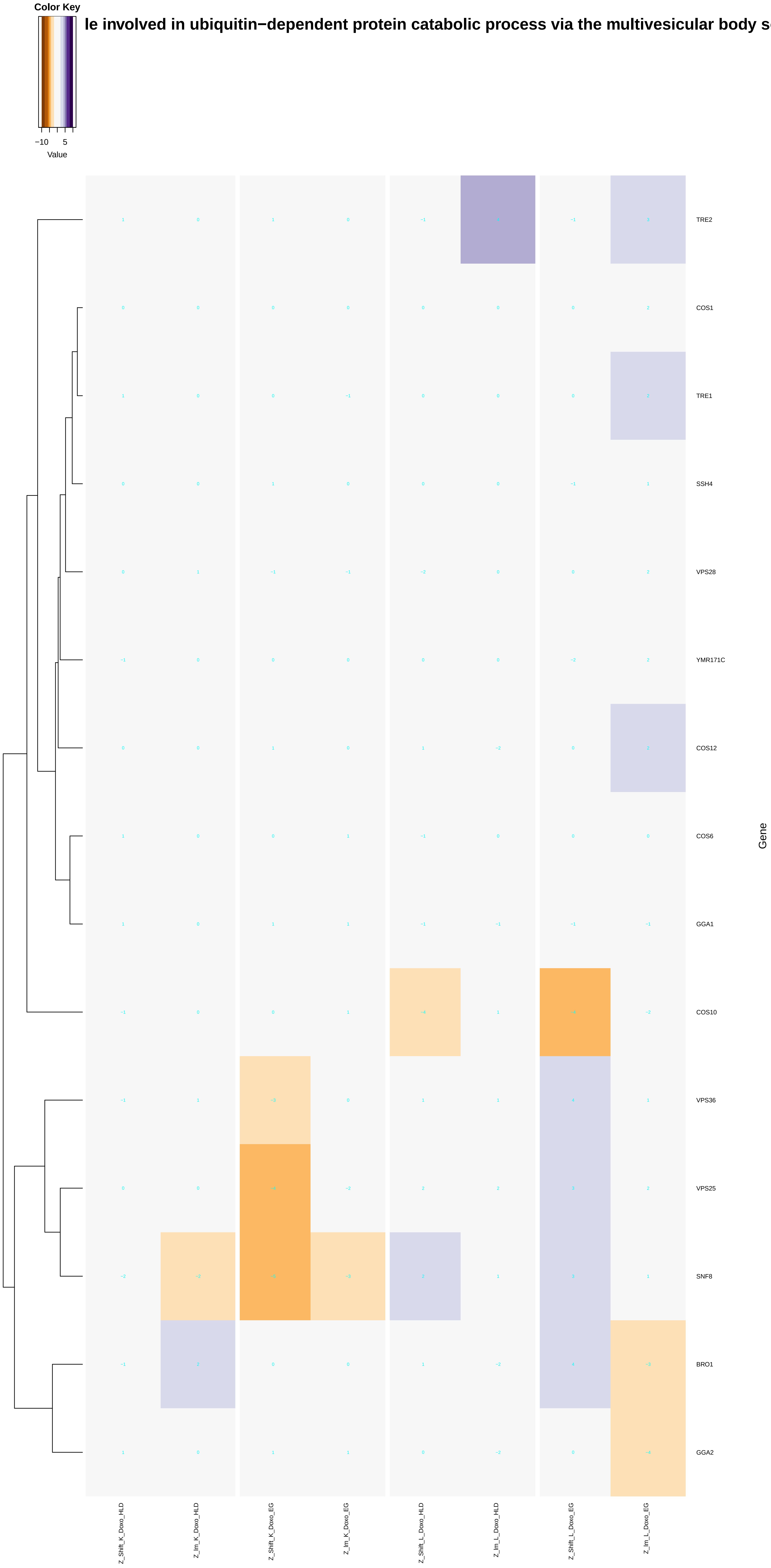

Color Key
−10
5
Value
le involved in ubiquitin−dependent protein catabolic process via the multivesicular body s
TRE2
COS1
TRE1
SSH4
VPS28
YMR171C
COS12
COS6
GGA1
COS10
VPS36
VPS25
SNF8
BRO1
GGA2
Gene
Z_Shift_K_Doxo_HLD
Z_Im_K_Doxo_HLD
Z_Shift_K_Doxo_EG
Z_Im_K_Doxo_EG
Z_Shift_L_Doxo_HLD
Z_Im_L_Doxo_HLD
Z_Shift_L_Doxo_EG
Z_Im_L_Doxo_EG

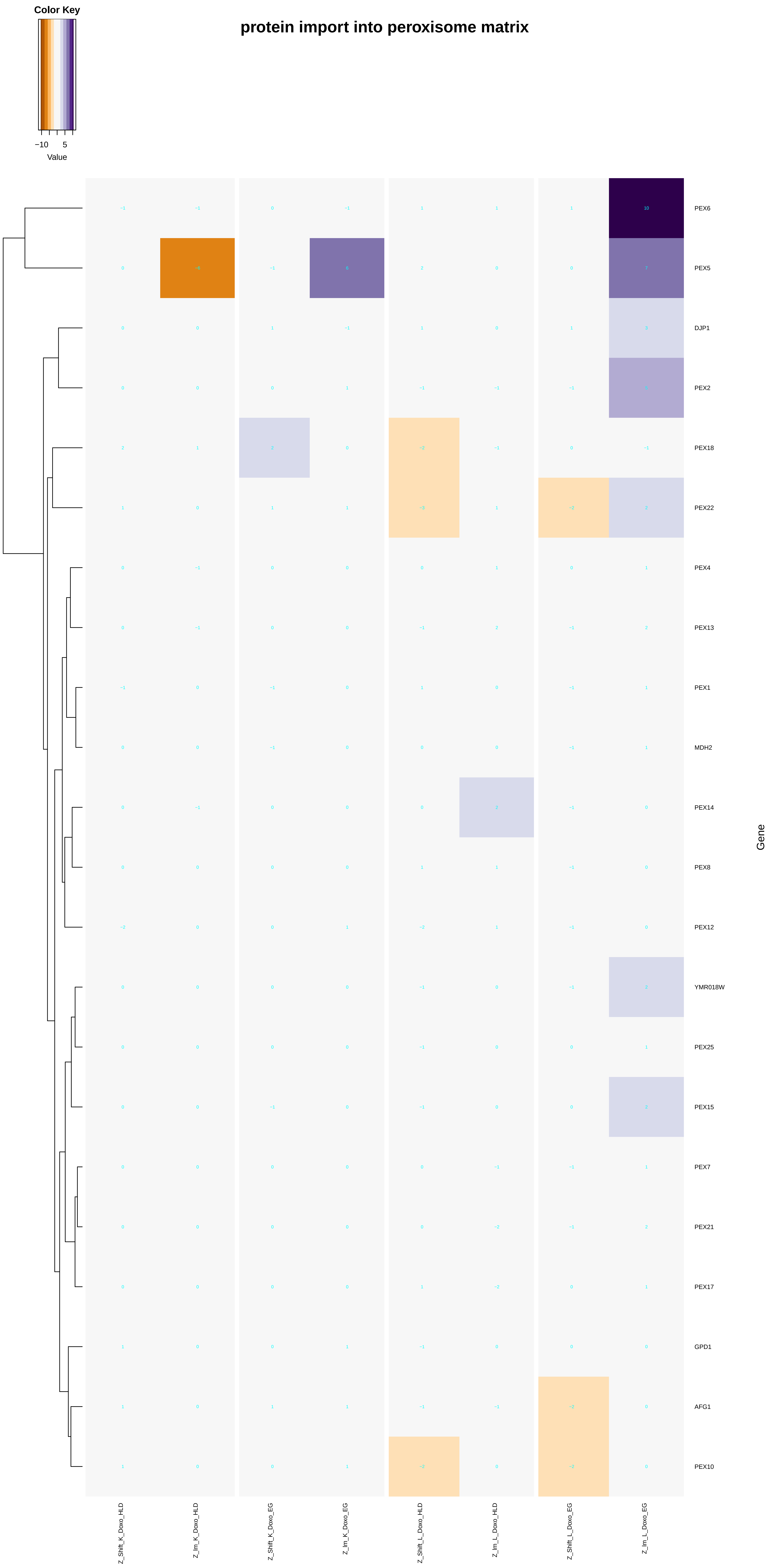
protein import into peroxisome matrix
Color Key
−10
5
Value
PEX6
PEX5
DJP1
PEX2
PEX18
PEX22
PEX4
PEX13
PEX1
MDH2
PEX14
PEX8
PEX12
YMR018W
PEX25
PEX15
PEX7
PEX21
PEX17
GPD1
AFG1
PEX10
Gene
Z_Shift_K_Doxo_HLD
Z_Im_K_Doxo_HLD
Z_Shift_K_Doxo_EG
Z_Im_K_Doxo_EG
Z_Shift_L_Doxo_HLD
Z_Im_L_Doxo_HLD
Z_Shift_L_Doxo_EG
Z_Im_L_Doxo_EG

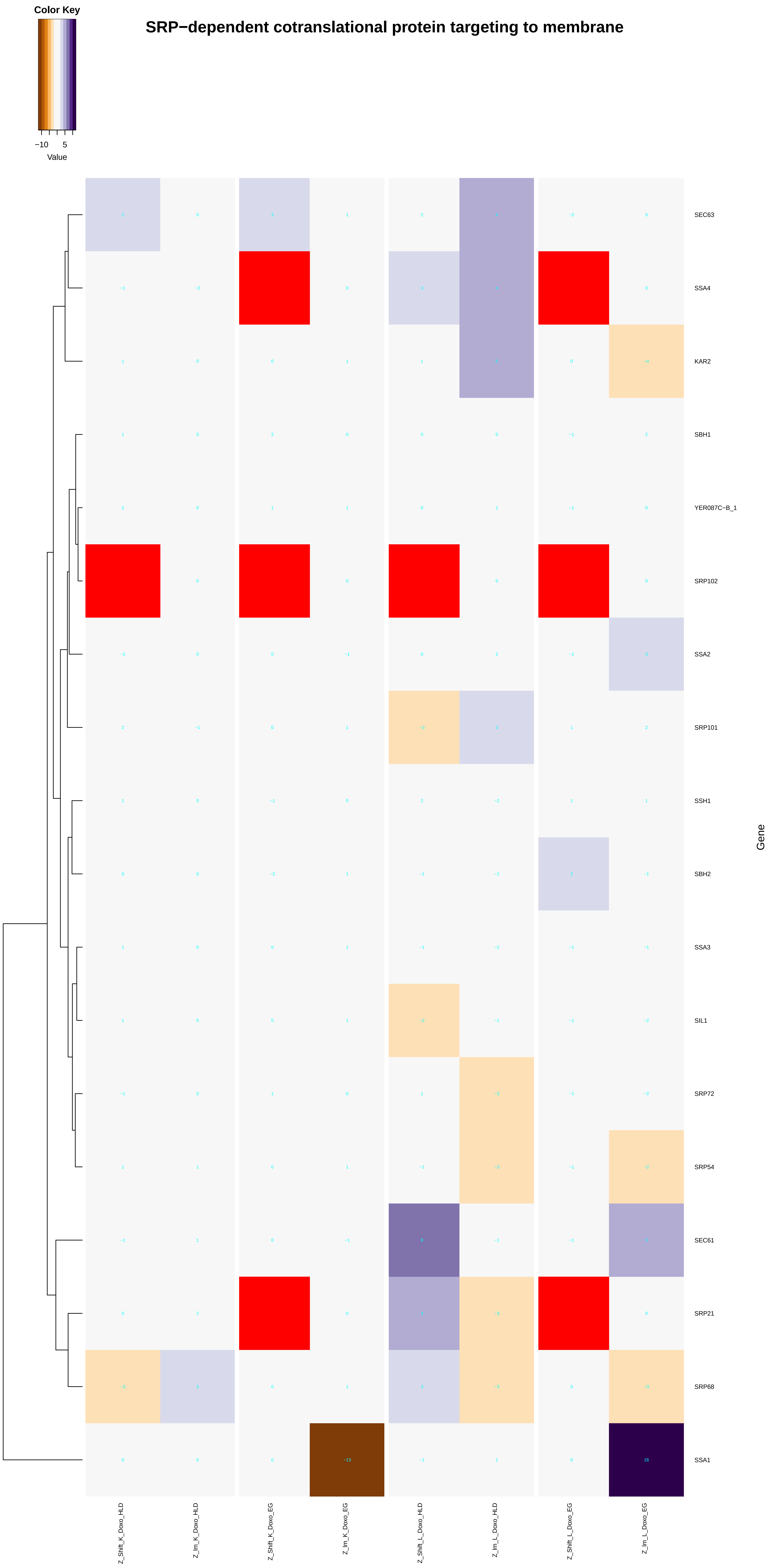

Color Key
−10
5
Value
SRP−dependent cotranslational protein targeting to membrane
SEC63
SSA4
KAR2
SBH1
YER087C−B_1
SRP102
SSA2
SRP101
SSH1
SBH2
SSA3
SIL1
SRP72
SRP54
SEC61
SRP21
SRP68
SSA1
Gene
Z_Shift_K_Doxo_HLD
Z_lm_K_Doxo_HLD
Z_Shift_K_Doxo_EG
Z_lm_K_Doxo_EG
Z_Shift_L_Doxo_HLD
Z_lm_L_Doxo_HLD
Z_Shift_L_Doxo_EG
Z_lm_L_Doxo_EG
